# Mental health clustering and diagnosis in psychiatric in-patients

**DOI:** 10.1192/pb.bp.114.047043

**Published:** 2015-06

**Authors:** Liam Trevithick, Jon Painter, Patrick Keown

**Affiliations:** 1 Northumberland, Tyne and Wear NHS Foundation Trust, UK

## Abstract

**Aims and method** This paper investigates the relationship between cluster (Mental Health Clustering Tool, MHCT) and diagnosis in an in-patient population. We analysed the diagnostic make-up of each cluster and the clinical utility of the diagnostic advice in the Department of Health’s *Mental Health Clustering Booklet*. In-patients discharged from working-age adult and older people’s services of a National Health Service trust over 1 year were included. Cluster on admission was compared with primary diagnosis on discharge.

**Results** Organic, schizophreniform, anxiety disorder and personality disorders aligned to one superclass cluster. Alcohol and substance misuse, and mood disorders distributed evenly across psychosis and non-psychosis superclass clusters. Two-thirds of diagnoses fell within the MHCT ‘likely’ group and a tenth into the ‘unlikely’ group.

**Clinical implications** Cluster and diagnosis are best viewed as complimentary systems to describe an individual’s needs. Improvements are suggested to the MHCT diagnostic advice in in-patient settings. Substance misuse and affective disorders have a more complex distribution between superclass clusters than all other broad diagnostic groups.

A set of needs-based clusters were originally developed as a classification system to aid service improvement in secondary care mental health services.^1^ There are a total of 21 clusters grouped into three superclasses: non-psychosis, psychosis and organic. Each cluster describes a particular type, combination and severity of needs. This Care Pathways and Packages approach (www.cppconsortium.nhs.uk) was subsequently adopted as the heart of a move away from block contracts towards a new mental health payment system.

The first 12 scales of the Mental Health Clustering Tool (MHCT) are Health of the Nation Outcome Scales (HoNOS) items.^2^ The HoNOS tool was originally developed by the Royal College of Psychiatrists’ Research Unit as an outcome measure. The Care Pathways and Packages Project developed the remaining six MHCT scales to support the classification of patients based on their level of need.^1^ The *Mental Health Clustering Booklet* provides likely diagnoses for each cluster. Added to version 3.0 of the booklet were unlikely diagnoses for each cluster based on the ICD-10 classification system used in the National Health Service (NHS).^3,4^

## Aims

The study had two aims. The first was to analyse the diagnostic make-up of each cluster from an in-patient population and to investigate the clinical utility of the advice in the MHCT booklet regarding likely and unlikely diagnoses. The second aim was to investigate the distribution of diagnoses across both superclass cluster groups and individual clusters.

## Method

All discharges from working-age adult or older people’s in-patient services in Northumberland, Tyne and Wear NHS Foundation Trust (NTW) between 1 April 2012 and 31 March 2013 were included in the study (each discharge refers to one treatment episode rather than one patient). Working-age adult services included psychiatric intensive care units (PICUs), acute adult and rehabilitation wards. Older people’s wards included functional, organic and long-term/complex need wards.

The cluster on admission was taken as cluster assigned on date of admission or up to 3 days after admission, or nearest cluster prior to admission. Cluster superclass groups used are those given in the MHCT booklet: non-psychosis clusters 1–8; psychosis clusters 10–17; and organic clusters 18–21. Patients were assigned to cluster 0 if they were not adequately described by another cluster but required secondary care services.

We considered the diagnosis on discharge because diagnosis is more likely to be recorded on discharge and is more likely to be related to the current episode than diagnosis on or prior to admission. The ICD-10 code was sourced from electronic patient records. The broad diagnostic groups used were F00–09 (organic and dementia), F10–19 (substance misuse), F20–29 (schizophrenia and related disorders), F30–39 (affective disorders), F40–48 (neurotic, stress-related and somatoform disorders), F60–69 (personality and behaviour disorders), and ‘other’ diagnoses. ‘Other’ diagnoses included F50–59 (behavioural syndromes associated with physiological disturbances and physical factors), F70–79 (mental retardation), F80–89 (disorders of psychological development), other developmental disorders, G00–99, H00–95, R00–99, S00–T98 and Z00–99. The next diagnostic level used to analyse the data was at the ‘three character’ level, for example F32 depressive episode or F33 recurrent depressive disorder. Within affective disorder the four-character level was used to distinguish conditions with and without psychosis.

To empirically validate the MHCT ‘likely’ and ‘unlikely’ advice, a threshold of 65% was set for the cumulative proportion of discharges in a cluster which were attributed to the ‘likely’ diagnoses. In other words, all of the ‘likely’ diagnoses for a cluster should account for 65% or more of discharges. An acceptable threshold of 10% was set for the ‘unlikely’ diagnoses, meaning that all ‘unlikely’ diagnoses for a cluster should not account for more than 10% of discharges. Diagnoses not in the likely or unlikely groups were put in the ‘other’ group.

All analysis of the data was performed using SPSS version 19 for Windows and Microsoft Excel.

This study was part of a service evaluation of clinical activity in NTW which focused on the use of in-patient services across the trust. This was within the Transforming Services Programme which had approval of the trust board and included ongoing work to evaluate the use of MHCT within the trust. Data were provided by the trust informatics department. We used routinely collected data for this study.

## Results

In total, there were 2830 discharges between 1 April 2012 and 31 May 2013. Primary discharge diagnosis was available for three-quarters of these (*n* = 2094): half were accounted for by affective disorders (*n* = 552, 26%) and schizophrenia and related disorders (*n* = 533, 25%). The remaining diagnostic groups were: personality disorders (*n* = 283, 14%); alcohol and substance misuse (*n* = 260, 12%); neurotic, stress-related and somatoform disorders (*n* = 253, 12%); dementia and organic disorders (*n* = 131, 6%); other (*n* = 82, 4%).

Over 90% of discharges (*n* = 2570) had an admission cluster. Of those, almost half (45%, *n* = 1145) were non-psychosis clusters 1–8, 42% (*n* = 1091) were psychosis clusters 10–17, 11% (*n* = 287) were organic clusters 18–21 and 2% (*n* = 47) were cluster 0.

There were 1937 discharges with both a cluster on admission and diagnosis on discharge. The diagnostic make-up of clusters 1, 2 and 21 was not analysed due to low numbers. In 11 of the remaining 17 clusters, the likely diagnoses made up more than 65% of the cases. This figure was highest for clusters 13, 16 and 17 where the likely diagnoses accounted for more than 80% of the cases. In 6 of the 17 clusters the likely diagnoses accounted for less than 65% of the cases and made up half or less of cases in clusters 3, 4, 10, 15 and 18. These same five clusters had high rates of ‘other’ diagnoses.

In the majority of clusters the ‘unlikely’ diagnoses made up around 10% of cases. In four of the clusters in the psychosis superclass (clusters 11, 12, 15 and 16) the ‘unlikely’ diagnoses accounted for between 11 and 17%.

Highlighted in Table 1 are five clusters which have low numbers of ‘likely’ diagnoses and relatively high numbers of ‘other’ diagnoses. There were a number of unexpected diagnoses for some clusters, particularly within non-psychosis clusters. Alcohol misuse was the primary diagnosis in more than 10% of those in clusters 3, 4 and 5. Personality disorder was the primary diagnosis in more than 10% of cluster 3; similar figures were found for recurrent depression in clusters 6 and 15, and organic disorders (F04–09) in clusters 18 and 19. More than a third of those in cluster 15 had an F20–29 diagnosis. Alcohol and substance misuse was the primary diagnosis for a fifth of cluster 10, whereas depression accounted for 10%.

Table 2 shows the distributions of broad diagnostic groupings among the cluster superclasses. There was a significant relationship between cluster and diagnosis: F00–09 largely falling within the organic superclass; F20–29 largely falling within the psychosis superclass; F40–48 and F60–69 largely falling within the non-psychosis superclass.

Substance misuse and affective disorders were split between the psychosis and non-psychosis superclass clusters. Table 3 shows a significant relationship between different types of substance misuse and superclass. Two-thirds of F10 diagnoses fell in the non-psychosis supercluster and nearly half were classified as having alcohol dependence (F10.2). In contrast, 67% of the F11–18 diagnoses fell within the psychosis supercluster. Multiple substance misuse diagnoses were equally split between these two superclass clusters.

A marked distinction between mania and bipolar disorders and the remaining affective disorders was observed (Table 3). There was a non-significant trend towards clustering bipolar disorder in the psychosis clusters, regardless of whether the patient exhibited psychotic symptoms or not. Patients with depression were significantly more likely to be assigned to non-psychosis clusters than to psychosis clusters. The only exception to this was depression with psychosis (Table 3).

## Discussion

The results show that the diagnostic advice in the clustering booklet holds true for ‘likely’ diagnoses in 11 of the 17 clusters analysed and in 13 clusters for ‘unlikely’ diagnoses. In five clusters (3, 4, 10, 15 and 18) the ‘likely’ diagnoses accounted for half or less of discharges from hospital and there were particularly high rates of other diagnoses.

**Table 1:** Discharges from each cluster and the percentage in the likely, unlikely and other diagnoses from the Mental Health Clustering Tool advice. Individual clusters with low rates of ‘likely’ diagnosis and high rates of ‘other’ diagnoses in bold

		Diagnoses, %		
Cluster	Total number	Likely	Unlikely	Other
Non-psychosis (1–8)	896	59	9	32
1	4	N/A	N/A	N/A
2	17	N/A	N/A	N/A
**3**	**72**	**46**	**7**	**47**
**4**	**189**	**49**	**9**	**42**
5	151	68	7	25
6	67	75	9	16
7	157	65	10	25
8	239	62	10	28

Psychosis (10–17)	899	73	10	17
**10**	**133**	**52**	**0**	**48**
11	111	74	17	9
12	169	76	15	9
13	102	83	6	11
14	220	74	10	16
**15**	**28**	**29**	**11**	**60**
16	80	81	15	4
17	56	93	4	3

Organic (18–21)	113	66	3	31
**18**	**21**	**52**	**5**	**43**
19	50	68	0	32
20	32	78	0	22
21	10	N/A	N/A	N/A

Total	1908	66	9	25

**Table 2:** Broad ICD-10 diagnostic groups at discharge and superclass cluster group at admission

	Superclass cluster groups				
	Cluster 0	Non- psychosis clusters 1–8	Psychosis clusters 10–17	Organic clusters 18–21	Total
	
	*n* (%)				
F00–09 Dementia and organic disorders	5 (4)	10 (8)	14 (12)	92 (76)	121 (100)

F10–19 Substance misuse	3 (1)	128 (56)	93 (40)	6 (3)	230 (100)

F20–29 Schizophrenia and related disorders	6 (1)	31 (6)	445 (92)	2 (0)	484 (100)

F30–39 Affective disorders	7 (1)	255 (49)	254 (49)	1 (0)	517 (100)

F40–48 Neurotic, stress-related and somatoform disorders	3 (1)	199 (82)	40 (17)	0 (0)	242 (100)

F60–69 Personality and behaviour disorders	2 (1)	235 (87)	34 (13)	0 (0)	271 (100)

Other diagnoses	2 (3)	39 (54)	19 (26)	12 (17)	72 (100)

Missing diagnostic data	19 (3)	248 (39)	192 (30)	174 (27)	633 (100)

Total	47 (2)	1145 (45)	1091 (42)	287 (11)	2570 (100)
	
	χ^2^ = 1622.7, d.f. = 14, *n* = 2523, *P* < 0.001				

Caution must be taken when interpreting some of these findings due to low sample numbers in some of the clusters. Further analysis in both in-patient and out-patient populations is necessary. Our findings indicate that the diagnostic advice holds true for the majority of clusters. However, the low rates of ‘likely’ diagnoses among a few clusters suggest that the current advice for those clusters does not hold true for a subsection of the in-patient population. These findings are supported by previous research which found high rates of mismatch between ICD-10 diagnoses and clusters 3, 4, 15 and 18.^5^

Clinical practice issues could partly explain these findings, but if further in-depth analysis in other trusts reveals similar trends, then changing the ‘likely’ diagnosis advice will increase the MHCT booklet’ clinical usefulness. Our results indicate that the following diagnoses could be added to the ‘likely’ diagnoses group: alcohol misuse for clusters 3 to 5; recurrent depression for cluster 6 and 15;

**Table 3:** The distribution of F10–19 substance misuse and F30–39 affective disorder diagnoses across the non-psychosis and psychosis superclass groups. Cluster 0 and organic superclass are not shown separately, but are included in total numbers

	MHCT groups		
ICD-10 diagnostic group	Total cluster 1–8	Total cluster 10–17	Total
F10–19 alcohol and substance misuse	128 (56%)	93 (40%)	230 (100%)
F10 alcohol	80 (67%)	31 (26%)	119 (100%)
F11–18 opioids, cannabinoids, sedatives, stimulants etc.	7 (29%)	16 (67%)	24 (100%)
F19 multiple drug use	41 (47%)	46 (53%)	87 (100%)
		
	χ^2^ = 20.41, d.f. = 2, *n* = 221, *P* < 0.001		

F30–39 affective disorder	255 (49%)	254 (49%)	517 (100%)
F30–31 mania and bipolar disorder	49 (20%)	192 (78%)	245 (100%)
F32–33 depression	196 (76%)	59 (23%)	259 (100%)
F34–39 persistent mood disorders, other mood disorders and mood disorders unspecified	10 (77%)	3 (23%)	13 (100%)
		
	χ^2^ = 162.22, d.f. = 2, *n* = 509, *P* < 0.001		

F31 bipolar disorder			
with psychotic symptoms	8 (19%)	34 (79%)	43 (100%)
without psychotic symptoms	20 (29%)	48 (69%)	70 (100%)
mixed episode	2 (15%)	11 (85%)	13 (100%)
unspecified	16 (14%)	97 (85%)	114 (100%)
		
	χ^2^ = 6.46, d.f. = 2, *n* = 236, *P* = 0.09		

F32 and F33 depression			
with psychotic symptoms	22 (38%)	35 (60%)	58 (100%)
without psychotic symptoms	93 (86%)	13 (12%)	108 (100%)
unspecified	81 (88%)	11 (12%)	92 (100%)
		
	χ^2^ = 60.45, d.f. = 2, *n* = 255, *P* < 0.001		

schizophrenia and related disorders to cluster 15; organic conditions (F04–09) to clusters 18 to 21.

There were two diagnostic areas that are worth discussing further. The first was personality disorder. A number of diagnoses of personality disorder were found in cluster 8. However, there were also some found in other non-psychosis clusters including clusters 3 and 4. It may be that these were incorrectly diagnosed or clustered. Alternatively, it may be that those with complex and severe personality disorders are allocated to cluster 8 whereas those with simple personality disorders are clustered lower down within the non-psychosis superclass.^6^

A further area of concern was the high proportion of ‘other’ diagnoses in cluster 10, a substantial proportion of which were alcohol and substance misuse diagnoses. This is at odds with the finding that the prevalence of drug-induced psychosis is relatively low in England.^7^ It may reflect a reluctance of some early intervention psychosis services to give a formal diagnosis early on in an individual’s contact with services.^8^

Whereas dementia and organic disorders, neurotic and stress-related and somatoform disorders, and schizophrenia and related disorders aligned to one superclass cluster group, affective disorders and substance misuse disorders did not. At one diagnostic level down, depression mainly fell within the non-psychosis superclass while mania and bipolar disorder fell within the psychosis superclass. The only exception to this was the diagnosis of psychotic depression. Local trust policy, in line with Royal College of Psychiatrists advice, was to cluster patients with bipolar disorder to the psychosis clusters, regardless of whether psychotic symptoms were present.^9^

Department of Health guidance currently being drafted suggests that patients with bipolar disorder diagnoses may be allocated to either psychotic or non-psychotic clusters depending on presenting needs,^10^ supporting the view that cluster and diagnosis should best be viewed as complementary. These findings also have implications for the proposed reorganisation of services. If there is to be a division between psychosis and non-psychosis, it is evident that both teams will require expertise in the management of affective disorders.

Further analysis showed that F10–19 alcohol/substance misuse accounted for 14% of all clusters 2–8 and was largely uniform across each cluster individually. This highlights that patients with a primary diagnosis of alcohol/substance misuse experience a wide range of problems and have varying levels of need. This can be seen as supporting the previous decision to disaggregate the original generic substance misuse cluster 9.^11^

### Limitations

There are a number of limitations of this research which need to be highlighted. First, the accepted thresholds used for ‘likely’ and ‘unlikely’ diagnoses were set by the research team. No previous research was available in which to benchmark against. Second, only in-patient discharges were included, but the MHCT was developed for use in both community and in-patient services. To acquire a fuller understanding of the cluster–diagnosis relationship, the research should be extended to community patients. Third, for low-need non-psychosis and organic clusters in particular, there were low numbers, meaning that reliable and valid conclusions could not be drawn. Fourth, it is important to note that during the first analysis, we grouped together all ‘likely’ diagnoses and did not separate out the relative contributions of each diagnosis. It is possible that a ‘likely’ diagnosis occurred rarely and was offset by a more frequent one. Fifth, audits established that cluster accuracy for the period from January to June 2012 was at 68% (CAPITA, personal communication, 2013). However, assignment to superclass cluster was highly accurate; only one service user (2%) was assigned to the wrong supercluster. It is important to note that this audit was conducted using 63 patients who had been clustered to a psychosis cluster only. This suggests that findings of associations at the superclass level are likely to be more robust than at the individual cluster level. Finally, we used clinical diagnoses and due to the nature of the study it was not possible to check accuracy or interrater reliability.

This paper provides further information on the relationship between cluster and diagnosis in an in-patient setting. It supports the notion that cluster and diagnosis are best seen as complementary systems to describe an individual’s needs, rather than there being a 1:1 relationship. This particularly applies to affective and substance misuse disorders. The data identified different skill sets required for the management of in-patients in the psychosis, non-psychosis and organic clusters if services are to further specialise in these areas. Results suggest some of the interventions that would need to be delivered within these services. Future work should extend this research into community teams.
